# 

**Published:** 1878-05

**Authors:** 


DETROIT, April 18, 1878.

The next regular meeting of the American Medical College Association will be held in Buffalo, N. Y., beginning June 3d, at 10 A. M. Place of meeting to be in Medical College Rooms, Main street, corner of Virginia.

L. CONNOR, *Secretary and Treasurer.*

BALMANO SQUIRE, M. B., Lond., is the author of four essays upon skin diseases; 1, On Lupus Disease of the Skin and its Treatment by a new method; 2, On the Treatment of Chronic Eczema by Glycerole of the Subacetate of Lead; 3, On Port-Wine Mark, and its Obliteration without Scar; 4, On the Treatment of Psoriasis by an Ointment of Chrysophanic Acid. Numbers two and four have been received, and we abstract the following formulæ. R. Take of acetate of lead 5; litharge 3½; glycerine 20. Heat for half an hour in a boiling glycerine bath, constantly stirring, and filter in a gas-oven or other kind of heated compartments. The result is a perfectly clean and colourless liquid, of a somewhat more viscid consistency than pure glycerine." An ointment of the subacetate may be made; "glycerole of the subacetate of lead 6; vaseline 28; mix the two together." The form in which Squire prescribes chrysophanic acid, in psoriasis is:

℞. Chrysophanic acid, gr. v-ʒj:

Lard, . . . . . . . ℥j.

Digest the acid in the lard at the temperature of boiling water (i.e. in a water bath) for half an hour, stirring constantly, when "set" mix with pestle and mortar, adding a few drops of essential oil, with a view to scenting the ointment slightly."

Chrysophanic acid is the active principle of an old Eastern remedy known as Goa powder. The appendix contains the record of many successful cases of its use. He advises the employment of both the above mentioned agents.

THE POPULAR SCIENCE MONTHLY and its SUPPLEMENT for March and April, are of unusual interest. Spontaneous Generation, is discussed, pro and con by Dr. Bastian and Prof. Tyndall, in all its latest phases. Other articles of importance to Medical men are in the March number; Opium and its Antidote; Technical Education; Liquefaction of the Gases; and in the April number, the Eucalyptus in the Future, Review of Flint's, 'The Source of Muscular Power,' and Poisons of the Intelligence—Chloroform. No one who expects to keep up with the times upon scientific discoveries, can fail to subscribe for the *Popular Science Monthly.*

THE HOSPITAL GAZETTE AND ARCHIVES OF CLINICAL SURGERY is again changed, this time to a weekly, at the low price of $2,00 a year. Drs. Bermingham and Lyons remain as editors, and have associated with themselves Dr. H. H. Kane, as assistant. This change should be final, and it is to be hoped that the editors may receive sufficient approval to continue its publication. We wish them every success.

THE NORTH CAROLINA MEDICAL JOURNAL, Drs. M. J. De Rosset and T. F. Wood, editors, published at Wilmington, N. C., is full of instructive and scholarly productions, and the authors should be thanked for their efforts in giving the profession in North Carolina so excellent a periodical.

We welcome to our exchange list, the CANADIAN JOURNAL OF MEDICAL SCIENCE. Vol. III. No. 4, is at hand, and we find in it many practical articles upon Medicine, Surgery and Midwifery. The editors are Drs. M. Ogden, and R. Zimmerman,

The AMERICAN JOURNAL OF OBSTETRICS and Diseases of Women and Children,—quarterly—by Paul T. Mundé M, D. is received, and we especially notice Dr. T. G. Thomas's Article upon *Laparo-Elytrotomy:* a substitute for the Cæsarean Section. Five operations are reported, resulting in saving three mothers while four children were born alive. These are more favorable results than Cæsarean Section can show, and is due to the fact, no doubt, that the peritoneum is uninjured during the operation if skillfully performed. If the same success attends future trials, Laparo-Elytrotomy must entirely displace Cæsarean Section.

We have no space to mention the other articles which attracted our attention, and would refer the reader to them for his consideration.

ARCHIVES OF DERMATOLOGY, edited by L. Duncan Bulkley, A. M., M. D., and a large number of able collaborators. Vol. IV. No. II, contains a number of fine articles upon Syphilis, while its digest of late literature on skin diseases is complete and useful for reference.

THE AMERICAN JOURNAL OF THE MEDICAL SCIENCES. Dr. Hays uses so much discression in the selection of articles presented, and the care taken to expunge everything that is not scientific and practical, that he has brought this journal to so high an estate, that no words of ours can increase the esteem in which it is held. The present number, April 1878, is up to the standard, and is filled with valuable matter.

THE LONDON LANCET has been an authority in periodic Medical Literature for so long, that it is looked upon as almost infallible, and to those anxious to obtain glimpses of English thought, the reprint by Wm. C. Herald, New York, is ever welcome.

ATLAS OF SKIN DISEASES, by Louis A. Duhring, M. D. Part III. Philadelphia: J. B. Lippincott & Co., 1878. Price per part $2,50. The parts already published established for this work a high position which the present part more than sustains. Dermatology attracts so much attention at the present day, that every new production embodying advanced views, must gain the approval of the profession. Dr. Duhring is well known as an authority on skin diseases, and we hope that he may receive the financial favor which will allow him to continue the good work. Part III. contains four chromo-lithographs; 1. Eczema (Squamosum,) 2. Syphiloderma (Erythematosum,) 3. Purpura (Simplex,) 4. Syphiloderma, (Papulosum et Pustulosum.) They are all artistic in the highest degree, but especially so is the second; here the secondary syphilitic lesion is truthfully and accurately portrayed, and is nearly equal to a clinical presentation. The *Atlas* will ever remain a standard authority, and since the edition is limited, all who have the means should supply themselves with the series as published.

State Regulations of Vice, by Aaron M. Powell, New York: Wood & Holbrook, 1878, pp. 127. The scheme to license or regulate social vice, is one of the greatest importance to medical men, since no system has ever been found that did not rest for its effectiveness upon the medical examiner. This little work advocates the abolition of government regulation wherever founded, and strongly protests against its introduction into America.

Mortuary Experience of the Mutual Life Ins. Co. of New York, with tabulated reports, and an Analysis of the causes of death, by G. S. Winston, M. D., W. R. Gillette, M. D., and E. J. Marsh, M. D. Volume II. New York, 1878, pp. 224. Two years ago this Company published its mortuary experiences for thirty years, and they were found so useful that a second volume was ordered, more fully tabulating and detailing its statistics. The various causes of death are stated and analysed separately, and their result exhibited. Consumption is found to head the list, and its consideration occupies nearly half the entire volume. The effects of the presence or absence of hereditary family influences are ascertained and developed. Nativity, age, and residence are tabulated and conclusion made, bearing upon future selection of risks. Typhoid Fever and Apoplexy rank next as to cause of death, while Diseases of the Heart, Malarial Fever, Congestion of the Brain, and Cancer follow in succession. The volume shows care and industry in its preparation, and is a credit to its authors.

Transactions of the Thirty-Second Annual Meeting of the Ohio State Medical Society, held at Put-in-Bay, June 12, 13, and 14th, 1877: Cincinnati, 1877, pp. 200. J. H. Pooley presents an instructive paper upon Perityphlitic Abscess, with a table of 46 operations and their results, which is valuable as proving the safety of timely incision. The same writer in a memoir of Wm. M. Awl, M. D., mentions the fact, that the latter surgeon removed the parotid gland involving the ligature of the carotid artery. Dr. Muscroft takes strong grounds in asking for state regulations of vice, in a paper 'On the Prevention of the Spread of Syphilis.' The volume ends with a list of deceased and active members.

The Multum in Parvo Reference and Dose Book, by C. Henri Leonard, M. A., M. D. Third edition: Revised and enlarged; Detroit, 1878, pp. 150. Price 75c. Dr. Leonard is so well known from his *Vest-Pocket Anatomist*, and *Manual of Bardaging*, that this little volume only needs to be named for all to acknowledge its merits. It is unqualifiedly '*Multum in Parvo*,' containing a list of the doses of every medicine known to the profession, together with all their incompatibles and the poisons and their antidotes, and full tables of weights and measures. Under the head "Short Stops," can be found more useful information than in many large pretentious works.

### Books and Pamphlets Received.

The advantages and accidents of Artificial Anæsthesia, by Lawrence Turnbull, M. D. Philadelphia: Lindsay & Blakiston, 1878.

London *Lancet*, April 1878.

*Popular Science Monthly*, March and April, 1878, and Supplement No. XI. March, 1878.

Annual Report of the Rhode Island Hospital, 1878.

Clinical Thermometry in the Diseases of Women, by R. Ludlam, M. D. Philadelphia: Sherman & Co., 1877.

Lithotomy, by David Prince, M. D., of Jacksonville, Ill. Reprint from St. Louis *Medical and Surgical Journal*, April 1878.

### Contents of May Number.

### ORIGINAL.

American Medical College Association,........ 394

THE BUFFALO

### Medical and Surgical Journal

PUBLISHED MONTHLY,

Containing Original Articles, Reports of Medical Societies and Hospitals, Editorial, Reviews, Correspondence, Army News, etc., etc; including the usual variety of Medical Periodical Publications. Specimen copies sent on application.

Address Buffalo Medical & Surgical Journal, Buffalo, N. Y.

TERMS OF SUBSCRIPTION, $3.00 PER YEAR, IN ADVANCE.

All communications for publication shoud be sent before the fifteenth (15th) of each month, or they will of necessity lie over until the next number. No change can be made in advertisements after the twenty-fifth. Address, Buffalo Medical and Surgical Journal, 978 Main Street.

Correspondence and original articles are solicited. Publication is no evidence that the views of the author are endorsed.

